# Identifying potential biomarkers for type 2 diabetes in the adipose tissue of older adults via multiple machine learning algorithms

**DOI:** 10.1038/s41598-025-29141-9

**Published:** 2025-12-29

**Authors:** Yun-Sang Yu, Da Som Lee, Joo Hyun Lim, Yoo Jeong Lee

**Affiliations:** Division of Endocrine and Kidney Disease Research, Department of Chronic Disease Convergence Research, National Institute of Health, Cheongju, 28159 Korea

**Keywords:** Type 2 diabetes, Older adults, AIM2, FHOD3, LASSO regression, Machine learning, Biomarkers, Computational biology and bioinformatics

## Abstract

Age-related decline in adipose tissue function is closely associated with impaired insulin sensitivity and chronic low-grade inflammation, and these conditions contribute to type 2 diabetes (T2D) development in older adults. Therefore, reliable biomarkers may be helpful for early T2D diagnosis in older adults. We aimed to identify novel biomarkers linked to diabetes in older adults and to develop a predictive tool for diabetes diagnosis. We integrated transcriptomic analysis and machine learning to screen key genes associated with T2D in older adults. Gene expression datasets related to abdominal subcutaneous adipose tissue were obtained from the Gene Expression Omnibus (GEO) database. Through batch effect correction and differentially expressed gene (DEG) analysis of the combined dataset, 210 DEGs were identified. Functional enrichment analysis revealed that these DEGs were enriched mainly in inflammation- and immune-associated pathways. To extract T2D-predictive genes, we used three machine learning algorithms: LASSO, SVM-RFE and random forest. Two common genes, AIM2 and FHOD3, were consistently identified as the optimal biomarkers for distinguishing older adults with T2D from those without T2D. Receiver operating characteristic (ROC) curve analysis revealed high predictive performance. AIM2 and FHOD3 could serve as novel diagnostic and therapeutic targets for older adults with diabetes.

**Supplementary Information:**

The online version contains supplementary material available at 10.1038/s41598-025-29141-9.

## Introduction

Type 2 diabetes mellitus (T2D) is a growing global health concern, affecting approximately 462 million individuals in 2017, or 6.28% of the global population. Diabetes prevalence increases significantly with age, impacting 4.4% of those aged 15–49 years, 15% of those aged 50–69 years, and 22% of those aged 70 years and older, highlighting the heightened vulnerability of older adults to T2D and its associated complications. As the global population ages, the number of older adults living with T2D is expected to increase rapidly in the coming decades^1,2^. Consequently, the costs of diabetes treatment are also projected to increase substantially.

Adipose tissue, as a key energy reservoir and endocrine organ, plays a critical role in maintaining metabolic homeostasis, thereby influencing various metabolic disorders, including diabetes. Moreover, the adipose tissue plays a pivotal role in age-related metabolic dysfunction and longevity^3^. With aging, adipose tissue function deteriorates, leading to the development of insulin resistance, abnormal lipid deposition, and chronic inflammation. Specifically, aging induces a decline in adipocyte progenitor function and the accumulation of senescent cells, a potential source of aging-associated inflammation, triggering pathological pathways such as defective adipogenesis, aberrant adipocytokine production, and insulin resistance. Above all, aging adipose tissue is associated with chronic low-grade inflammation, known as inflammaging, which can promote other aging characteristics and affect the overall health status. Therefore, adipose tissue inflammaging is considered a potential therapeutic target in antiaging strategies^4^.

Age-related adipose tissue senescence is particularly prominent in subcutaneous adipose tissue (SAT) and are closely associated with systemic metabolic diseases. With age, macrophages are elevated in subcutaneous fat^5^ and telomere length is shorter in subcutaneous adipose tissue than in visceral adipose tissue^6^, which appears to be significantly associated with impaired glucose and lipid metabolism in adipose tissue. Furthermore, during the aging process, adipose tissue undergoes significant gene expression changes and is regulated by extracellular signaling molecules, contributing to metabolic disruption and the acceleration of aging. Age-related changes in adipose tissue, particularly in the context of diabetes, are not confined to local tissue alterations but have systemic implications, ultimately playing a crucial role in the onset and progression of diabetes. Specifically, SAT dysfunction and its associated metabolic changes are emerging as key mechanisms in aging-related diabetes. Understanding the relationship between adipose tissue aging and diabetes may provide valuable insights for preventing and treating age-related diseases, highlighting the need for further research in this area^3,7^.

In recent years, the integration of bioinformatics and machine learning algorithms has become a crucial approach for disease risk prediction, significantly advancing the ability to identify diagnostic biomarkers for various conditions. The rapid development of bioinformatics, alongside the application of machine learning to medical big data, has enabled the efficient screening of genes associated with disease onset and progression, offering faster and more accurate predictions than traditional experimental methods do^8,9^.

In this study, we applied advanced machine learning techniques, including least absolute shrinkage and selection operator (LASSO) regression^10^, support vector machine recursive feature elimination (SVM-RFE)^11^, and random forest (RF)^12^, to identify age-specific biomarkers in SAT. By integrating these approaches with transcriptomic data analysis, we aimed to pinpoint key genes uniquely associated with aging in SAT, shedding light on the potential roles of these genes in metabolic dysregulation and age-related diseases. Through this integrative approach, we obtained a novel perspective on the molecular mechanisms underlying SAT aging, thereby establishing a foundation for further precision medicine research targeting age-associated metabolic disorders.

## Methods

### Data processing and differentially expressed gene analyses

Transcriptomic datasets were obtained from the Gene Expression Omnibus (GEO, https://www.ncbi.nlm.nih.gov/geo/) database to identify specific age-related biomarkers in abdominal SAT from older adults with T2D. The selected datasets were GSE141432, GSE166047, and GSE175495, and they include comprehensive gene expression profiles and associated metadata. Raw count matrices and the human genome annotation table provided by the National Center for Biotechnology Information (NCBI) were utilized for this study. From each dataset, we selected samples corresponding to abdominal SAT that fit the criteria for two groups: the older healthy (OH) group and the older with type 2 diabetes (OT2D) group. Detailed information about the datasets is provided in Table 1. Normalization and differential gene expression analysis between the control group (OH) and the T2D group (OT2D) in the combined dataset were performed using DESeq2^13^. Combat-Seq from the sva package was used to correct for batch effects. The DEGs were identified on the basis of a log2-fold change threshold (|log2FC| ≥ 2) and an adjusted p value (padj < 0.05) as the criteria.

**Table 1:** Characteristics of the datasets used in the analysis.

GEO accession	GSE175495	GSE141432		GSE166047
	Older healthy (OH)		Older with type 2 diabetes (OT2D)	
Number of sample(n)	12	9	8	5
Age (years)	66.0 ± 5.0	62.0 ± 7.41	59.5 ± 5.93	54.6 ± 14.9
BMI (kg/m^2^)	24.8 ± 1.4	23.75 ± 1.04	33.75 ± 3.11	38.1 ± 11.8
Fat mass (kg)	18.8 ± 2.7	19.5 ± 5.93	34.75 ± 15.56	54.51 ± 12.39
Systolic blood pressure (mmHg)	132.0 ± 18.0	136.0 ± 18.52	145.0 ± 9.63	128.0 ± 4.47
Diastolic blood pressure (mmHg)	87.0 ± 7.0	80.0 ± 13.33	84.0 ± 6.67	80.0 ± 0.00
HbA1c (%)	–	5.27 ± 0.27	6.71 ± 0.95	–
HOMA-IR	1.47 ± 0.41	1.2 ± 0.44	2.4 ± 2.3	–

### Functional enrichment analysis

The selected DEGs were subjected to functional enrichment analysis via the enrichGO and enrichKEGG functions in ClusterProfiler for Gene Ontology (GO) and Kyoto Encyclopedia of Genes and Genomes (KEGG) enrichment analysis, respectively^14–17^. GO enrichment was categorized into three domains: biological process (BP), molecular function (MF), and cellular component (CC).

### Machine learning analysis

To identify candidate genes, we applied three machine learning algorithms: LASSO logistic regression, support vector machine–recursive feature elimination (SVM-RFE), and random forest (RF). The combined dataset was randomly divided into a training set (65%, *n* = 20) and a test set (35%, *n* = 11), with a fixed random seed (set.seed(100)) applied to ensure reproducibility.

LASSO logistic regression was performed using the “glmnet” package^18^ in R. The optimal regularization parameter (lambda.min = 0.01307997) was selected via 10-fold cross-validation^18^. SVM-RFE was implemented using the “caret^20^,” “e1071^21^,” and “kernlab^22^” packages in R. The model used a radial basis function (RBF) kernel with hyperparameters set to cost = 0.5 and gamma = 0.004400041, which were determined on the basis of performance optimization^19^. Random forest analysis was conducted using the “randomForest” package^24^ in R, with the number of trees set to 500 (ntree = 500). Model performance was evaluated via the built-in out-of-bag (OOB) error estimate, and gene importance was ranked on the basis of mean decrease in the Gini index^12^. Finally, the intersecting genes identified by all three algorithms (LASSO, SVM-RFE, and RF) were designated as hub genes and visualized via Venn diagrams.

### Construction and validation of the nomogram

The diagnostic performance of the hub genes was further evaluated by conducting receiver operating characteristic (ROC) curve analysis and computing the area under the ROC curve (AUC) using the pROC package^20^. The nomogram model was constructed using the rms package in R to visually represent the relative contributions of the selected genes within the diagnostic model, thereby clarifying the weight each gene carries in the overall prediction. Calibration curve analysis was performed to assess the agreement between the predicted and actual values, and 1000 bootstrapping steps were applied to validate the reliability of the model^21^.

### Animals

Male C57BL/6 mice (3 and 18 months old, *n* = 10–12 per group) were obtained from the Animal Facility of Aging Science, Korea Basic Science Institute (KBSI) Gwangju Center (Gwangju, Korea) and maintained under specific pathogen-free facility (SPF) conditions at a constant room temperature (RT; 23 °C) on a 12 h light/dark cycle with free access to food and water. Old male mice were caged individually. After 1 week of adaptation, young and aged mice were randomly assigned to two groups and were fed either normal chow diet or high-fat diet (60 kcal% fat) for 4 months. The body weights of the mice in each group were determined using an animal weight balance. Mice were euthanized by carbon dioxide (CO_2_) inhalation, and tissues were rapidly collected and snap-frozen in liquid nitrogen. All the animal experimental procedures were approved and followed the guidelines of the Institutional Animal Care and Use Committee of Korea National Institute of Health (permit number: KCDC-032-20-2 A). This study was conducted in compliance with the ARRIVE guidelines and regulations for the use of animals in research.

### Reverse transcription (RT)-quantitative PCR (RT‒qPCR)

Total RNA was extracted from gonadal fat pad using the RNeasy Mini Kit (Qiagen, USA) according to the manufacturer’s instructions. cDNA was synthesized via SuperScript IV reverse transcriptase (Invitrogen; Thermo Fisher Scientific, Inc.) and quantitative PCR was performed via the use of SYBR Green Master Mix (Thermo Fisher Scientific) in a total volume of 20 µL with the QuantStudio 6 Flex system (Thermo Fisher Scientific). The expression of the target genes was normalized to Gapdh expression, and relative quantification was performed with the 2^−ΔΔCt^ method.

### Statistical analysis

All the statistical analyses were conducted using R software (version 4.4.0.) and GraphPad Prism version 10 software (GraphPad, San Diego, CA, USA, http://www.graphpad.com). Comparisons between two groups of continuous variables with a normal distribution were performed via Student’s t test, whereas comparisons of nonnormally distributed variables were conducted using the Wilcoxon rank-sum test. For multiple-group comparisons, we checked whether the data from each group follows a normal distribution using the Shapiro-Wilk test method and the Kolmogorov-Smirnov test method. In case of normality, we performed a parametric one-way ANOVA with Tukey post-hoc test for multiple comparisons to assess the significance among pairs of conditions, whereas, in case of non-normality, we performed a Kruskal-Wallis test and a non-parametric post-hoc test. Values of *p* < 0.05 were considered statistically significant.

## Results

### Screening for DEGs

**Fig. 1:**
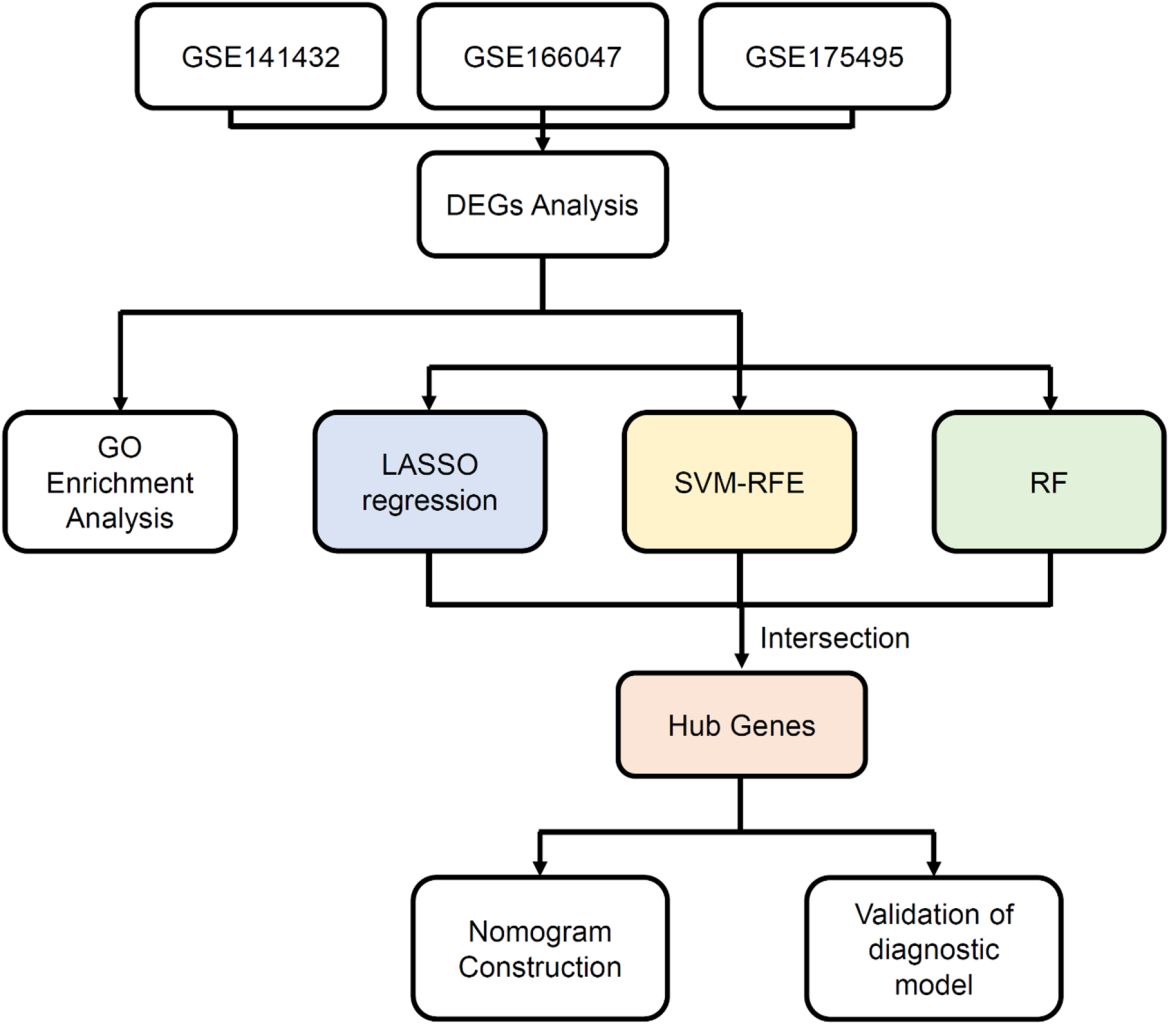
Flowchart of the study. *DEGs* Differentially expressed genes, *GO* Gene Ontology, *LASSO* least absolute shrinkage and selection operator, *SVM-RFE* support vector machine-recursive feature elimination, *RF* random forest.

**Fig. 2:**
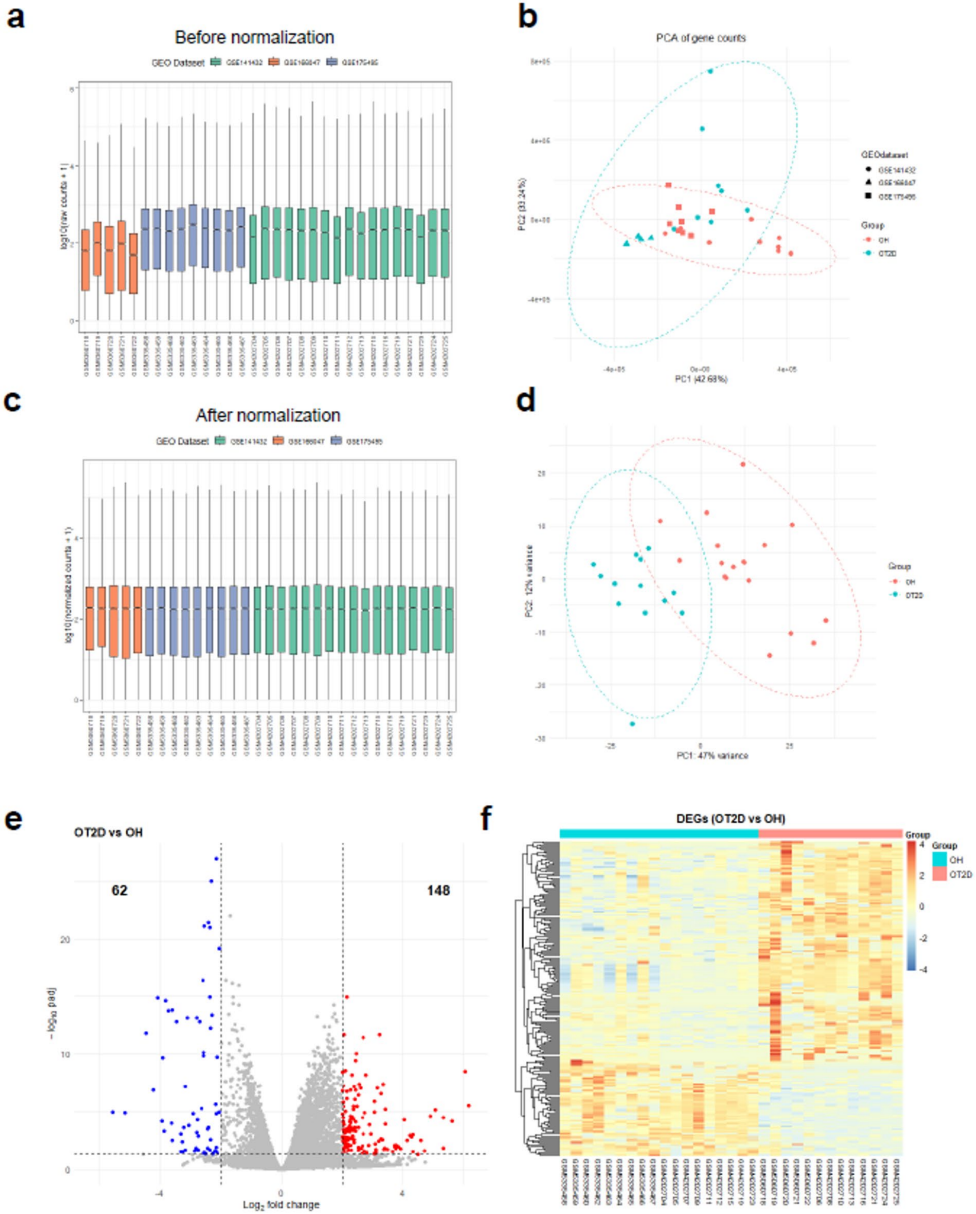
Normalization and identification of significant DEGs. (**A**) Box plot of the read count data and (**B**) Principal components analysis plot before normalization. (**C**) Boxplots of the normalized read count data and (**D**) PCA plot of vst-transformed data from three GEO datasets after differential expression analysis using DESeq2. (**E**) Volcano plots of DEGs between different groups highlighting genes that were significantly upregulated (red) or downregulated (blue) (padj < 0.05, |logFC| ≥ 2). (**F**) Heatmap analysis of significant DEGs. *DEGs* Differentially expressed genes, *PCA* principal component analysis, *GEO* Gene Expression Omnibus, *padj* djusted p value, *FC* functional change.

The flow chart of the detailed analysis process is shown in Fig. 1. First, we combined three expression array datasets (GSE141432, GSE166047, and GSE175495) obtained from the GEO database into a single discovery dataset. Because of the different backgrounds of the samples, the GEO data were normalized (Fig. 2A–C), and then, batch effects were removed via the sva package. Principal component analysis (PCA) revealed strong similarity among samples and tight clustering within both the OH group and the OT2D group (Fig. 2D). Afterward, the DEGs were screened according to the screening cutoff criteria padj < 0.05 and |logFC| ≥ 2. As a result, 210 genes were identified as being differentially expressed in OT2D, with 148 upregulated and 62 downregulated genes (Table S1−2), and the DEGs were visualized with volcano plots (Fig. 2E) and heatmap (Fig. 2F).

### Enrichment analysis of DEGs

**Fig. 3:**
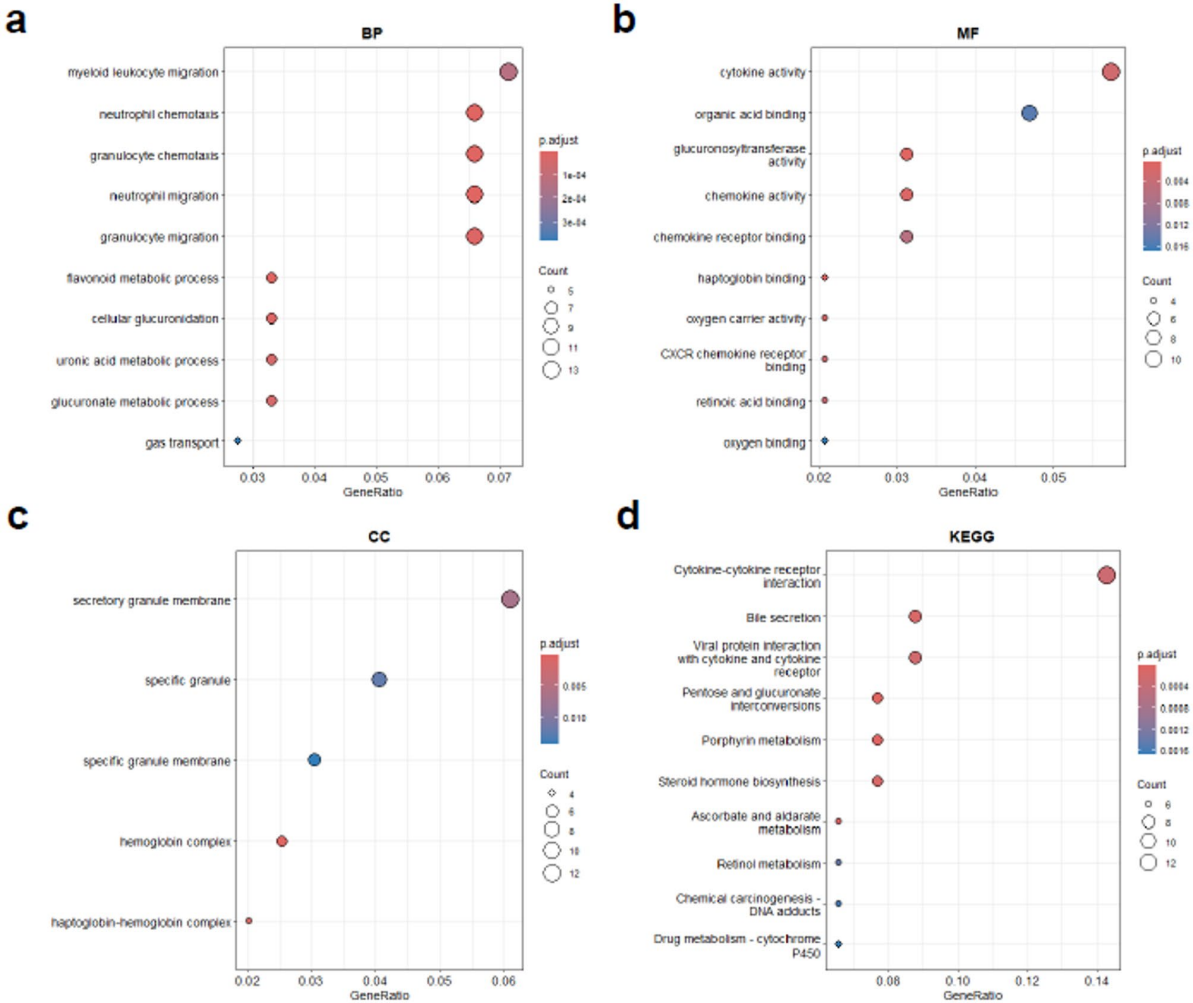
Functional enrichment analysis of DEGs. (**A**–**C**) GO functional enrichment analysis bubble chart for DEGs. (**D**) KEGG enrichment analysis of DEGs. *DEGs* Differentially expressed genes, *FC* functional change, *BP* biological process, *CC* cellular component, *MF* molecular function, *KEGG* Kyoto Encyclopedia of Genes and Genomes.

GO enrichment analysis revealed significant enrichment of these DEGs in various BPs, CCs, and MFs. In the BP category, the DEGs were involved primarily in myeloid leukocyte migration, neutrophil chemotaxis, granulocyte migration, and gas transport, highlighting the role of these DEGs in immune cell trafficking and oxygen transport. Additionally, genes associated with flavonoid metabolic processes, cellular glucuronidation, and uronic acid metabolism were enriched, suggesting potential metabolic adaptations. In the CC category, enrichment was observed for secretory granule membrane, specific granule membrane, hemoglobin complex, and haptoglobin-hemoglobin complex, indicating the involvement of these genes in immune granule secretion and hemoglobin-associated processes. The MF category included DEGs related to cytokine activity, chemokine receptor binding, oxygen carrier activity, and glucuronosyltransferase activity, emphasizing the roles of these DEGs in immune regulation, molecular transport, and metabolic modifications (Fig. 3A–C). Furthermore, KEGG pathway analysis revealed that the DEGs were involved primarily in immune regulation pathways, such as cytokine–cytokine receptor interactions and viral protein interactions with cytokines and cytokine receptors, underscoring the roles of these DEGs in inflammatory responses and immune modulation. Additionally, metabolic pathways, including steroid hormone biosynthesis and retinol metabolism, were enriched, suggesting a potential link to metabolic regulation. Moreover, pathways related to xenobiotic metabolism, such as drug metabolism – cytochrome P450, were also identified, indicating potential interactions with environmental or pharmacological factors (Fig. 3D).

### Identification of T2D diagnostic biomarkers via machine learning approaches

**Fig. 4:**
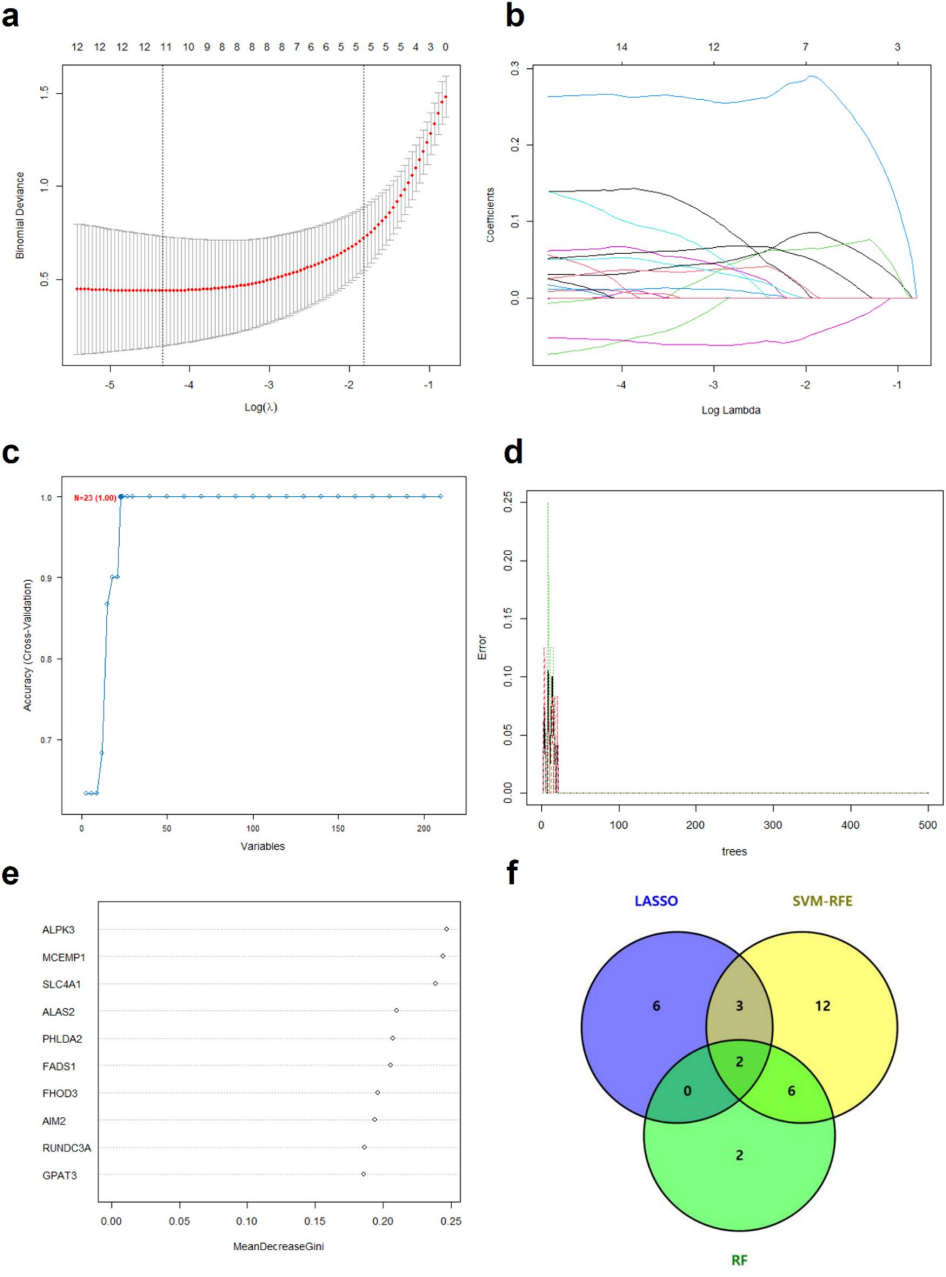
Machine learning-based screening of candidate diagnostic biomarkers for T2D in older adults. (**A**) Cross-validation plot for selecting the optimal tuning parameter log (Lambda) in LASSO regression. (**B**) LASSO coefficient profiles of candidate genes. (**C**) Feature selection via SVM-RFE. (**D**) RF error rate plot for the initially screened candidate genes. (**E**) Variable importance scores from the RF model, showing the top 10 genes. (**F**) Venn diagram of the LASSO, SVM-RFE and RF results. *T2D* Type 2 diabetes, *LASSO* least absolute shrinkage and selection operator, *SVM-RFE* support vector machine-recursive feature elimination, *RF* random forest.

To further identify potential diagnostic biomarkers of T2D in older adults, we applied three machine learning algorithms: LASSO regression, SVM-RFE, and RF. For this purpose, we randomly selected 65% of the total samples from each group (OH and OT2D) to construct the training set, while the remaining 35% of the samples were used as the test set for model evaluation. First, we applied LASSO regression to identify key biomarkers that distinguish the OH group from the OT2D group. Using 10-fold cross-validation, we determined the optimal lambda value (λmin), enabling us to prevent overfitting while selecting significant genetic features. As a result, 11 genes were considered feature selections (Fig. 4A,B). We subsequently employed the SVM-RFE method to rank the most predictive features in the SVM model. After tuning the hyperparameters through 10-fold cross-validation, we identified 23 key genes that play crucial roles in differentiating the OH and OT2D groups (Fig. 4C). Additionally, we utilized the RF algorithm to assess the importance of genes. After training the model, we calculated feature importance on the basis of the mean decrease in the Gini index, and we selected the top 10 genes with the highest importance (Fig. 4D,E). The intersection of the candidate genes identified by the three algorithms is illustrated in the Venn diagram in Fig. 4F. Finally, AIM2 and FHOD3 were identified as hub genes for T2D (Table 2).

**Table 2:** Feature selected genes of LASSO, SVM-RFE, and RF analysis.

LASSO	SVM-RFE		RF	Hub Genes
*CD52*	AIM2*	FHOD3*	*ALPK3*	***AIM2***
*PROK1*	*AKR1B15*	*GLT1D1*	*MCEMP1*	***FHOD3***
AIM2*	*ALAS2*	*GPAT3*	*SLC4A1*	
*CHI3L1*	*ALPK3*	*IFIT1B*	*ALAS2*	
*UGT1A1*	*AQP9*	*LRRC3C*	*PHLDA2*	
*PNPLA1*	*BCL2A1*	*MCEMP1*	*FADS1*	
*STMN2*	*CA1*	*MEIKIN*	FHOD3*	
*CLEC12A*	*CD52*	*NFE2*	AIM2*	
*PPP4R4*	*CHI3L1*	*PHLDA2*	*RUNDC3A*	
*CCL18*	*CR1L*	*PLEK2*	*GPAT3*	
FHOD3*	*FADS1*	*PNPLA1*		
	*FASN*			

### Evaluation and diagnostic performance of the marker gene model

**Fig. 5:**
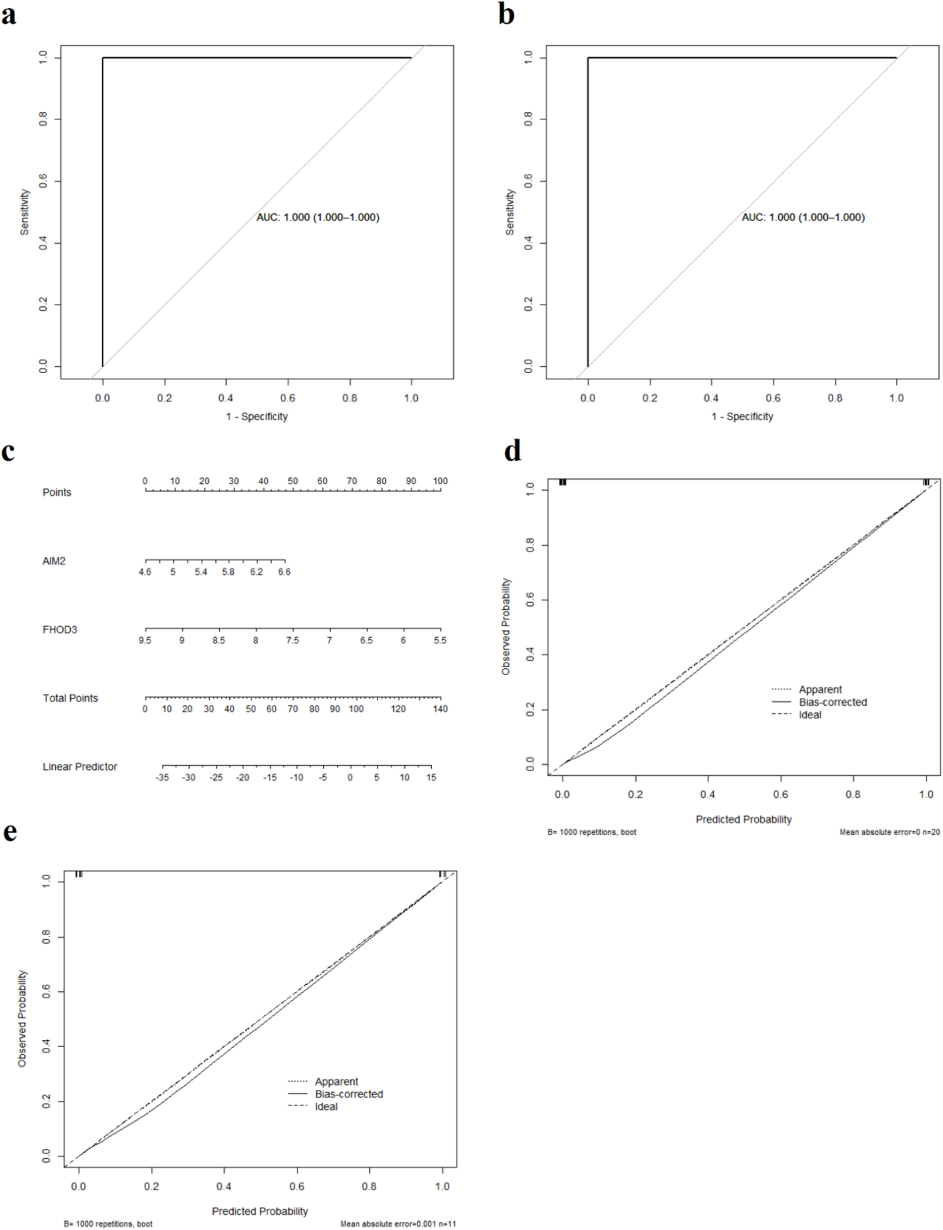
Evaluation of the marker gene model. (**A**) ROC curve for the hub genes in the training set. (**B**) ROC curve for the hub genes in the test set. (**C**) Nomogram diagnostic prediction model based on the hub genes. (**D**) Calibration curve of the marker gene model in the training set. (**E**) Calibration curve of the marker gene model in the test set. *ROC* Receiver operating characteristic.

To evaluate the predictive performance of the hub genes AIM2 and FHOD3, we conducted ROC curve analysis and nomogram-based assessment. In the training set, the model exhibited excellent discriminative ability, with an AUC of 1.000 (95% confidence interval (CI): 1.000–1.000) (Fig. 5A). Similarly, in the test set, the model maintained an AUC of 1.000 (95% CI: 1.000–1.000), indicating good diagnostic efficacy (Fig. 5B). In addition, we developed a nomogram to visualize the prediction model (Fig. 5C). Calibration curves of the nomogram confirmed the reliability of the model, with a C-index of 1.000 and a robust C-index of 1.000 in the training set (Fig. 5D). In the test set, the C-index remained at 1.000, whereas the robust C-index was slightly lower at 0.998 (Fig. 5E), indicating that the two biomarkers play pivotal roles in the diagnosis of T2D.

### Validation of the marker genes screened by machine learning approaches

**Fig. 6:**
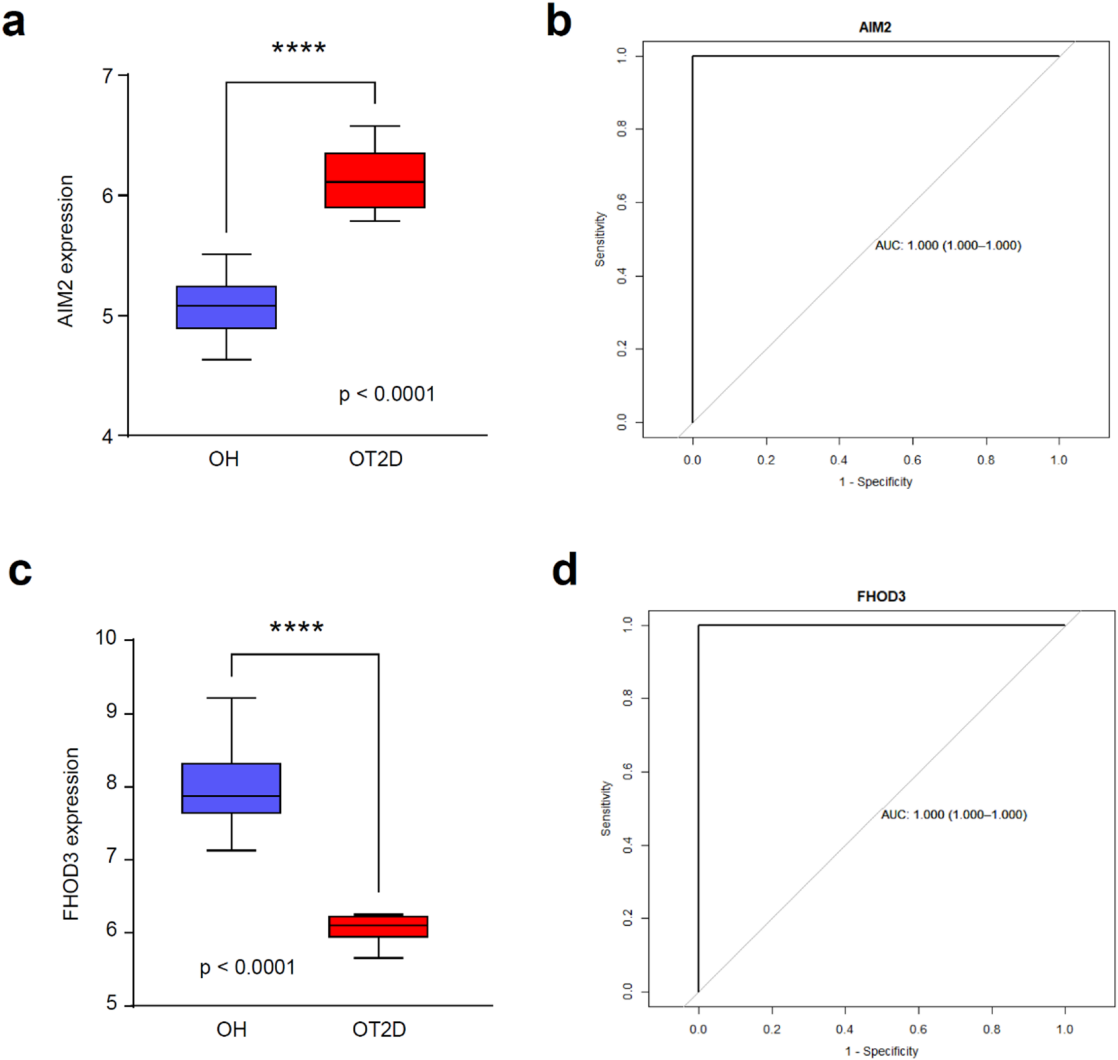
Expression and diagnostic performance of AIM2 and FHOD3 in the training set. (**A**) Expression levels of AIM2 in the OH and OT2D groups. (**B**) ROC curve of AIM2. (**C**) FHOD3 expression levels in the OH and OT2D groups. (**D**) ROC curve for FHOD3. *OH* Older healthy, *OT2D* older with type 2 diabetes, *ROC* receiver operating characteristic. *p-value < 0.05; ** *p-*value < 0.01; *** *p-*value < 0.001; **** *p-*value < 0.0001.

To further assess the diagnostic potential of individual marker genes, we analyzed the expression and classification performance of AIM2 and FHOD3 in both the training and test sets. In the training set, AIM2 expression was significantly upregulated in the OT2D group compared with the OH group (Fig. 6A). Afterward, we conducted ROC curve analysis to assess the diagnostic performance of AIM2, and the AUC was 1.000 (95% CI: 1.000–1.000) (Fig. 6B). Similarly, FHOD3 expression was significantly altered between the two groups (Fig. 6C), and ROC curve analysis revealed an AUC of 1.000 (95% CI: 1.000–1.000) (Fig. 6D). These findings indicate that higher expression of AIM2 and lower expression of FHOD3 may increase the risk of T2D in older adults.

**Fig. 7:**
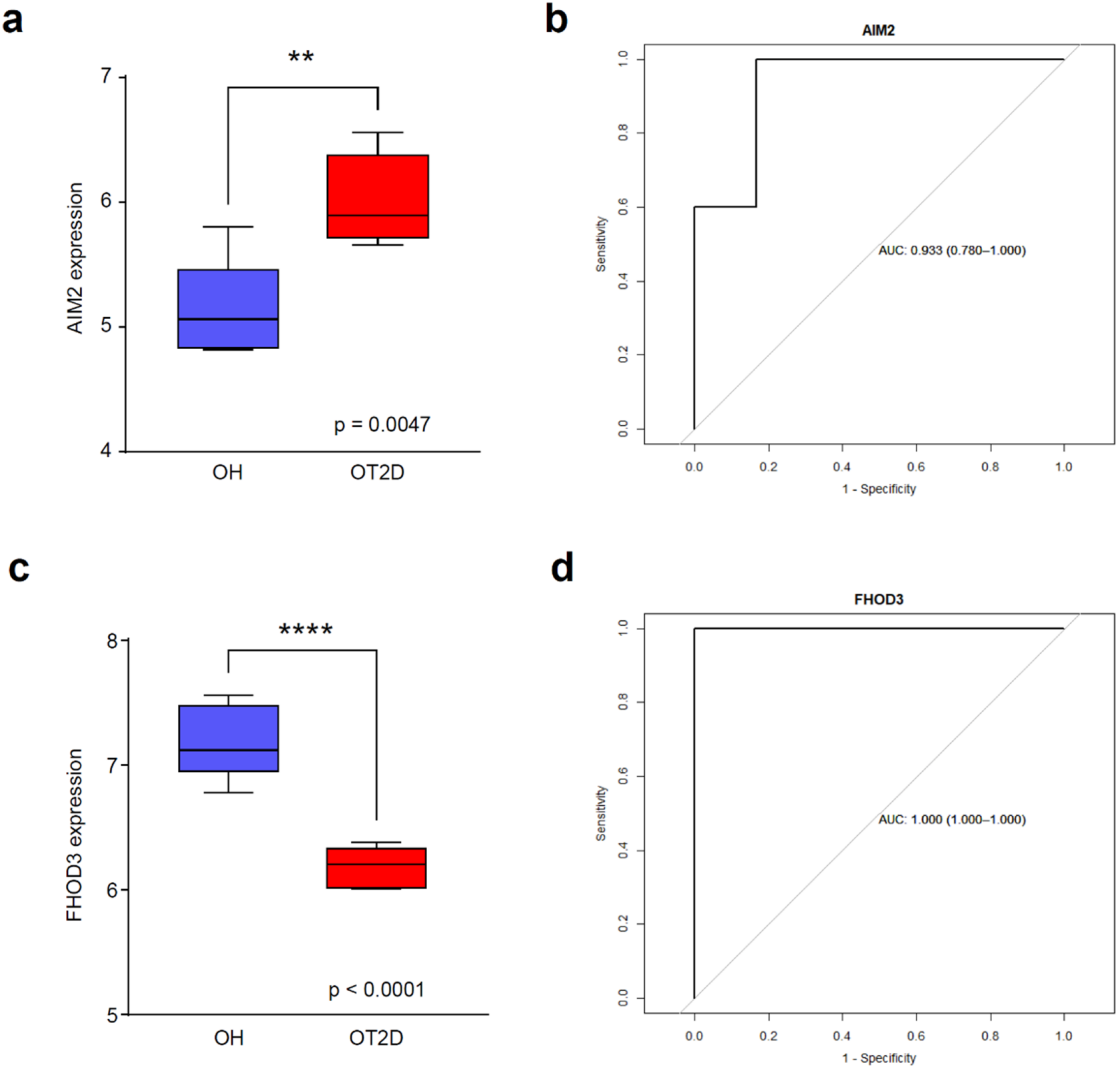
Expression and diagnostic performance of AIM2 and FHOD3 in the test set. (**A**) Expression levels of AIM2 in the OH and OT2D groups. (**B**) ROC curve of AIM2. (**C**) FHOD3 expression levels in the OH and OT2D groups. (**D**) ROC curve for FHOD3. *OH* Older healthy, *OT2D* older with type 2 diabetes, *ROC* receiver operating characteristic. *p-value < 0.05; ** *p-*value < 0.01; *** *p-*value < 0.001; **** *p-*value < 0.0001.

A similar pattern was observed in the test set. AIM2 and FHOD3 expression levels were significantly different between the OH and OT2D groups (Fig. 7A,C). The ROC curve analysis of AIM2 yielded an AUC of 0.933 (95% CI: 0.780–1.000) (Fig. 7B), whereas that of FHOD3 yielded an AUC of 1.000 (95% CI: 1.000–1.000) (Fig. 7D), confirming its diagnostic potential. Overall, these results collectively indicate that AIM2 and FHOD3 may serve as biomarkers for distinguishing elderly patients with T2D from those without T2D.

### Experimental validation of the expression of hub genes in mice

**Fig. 8:**
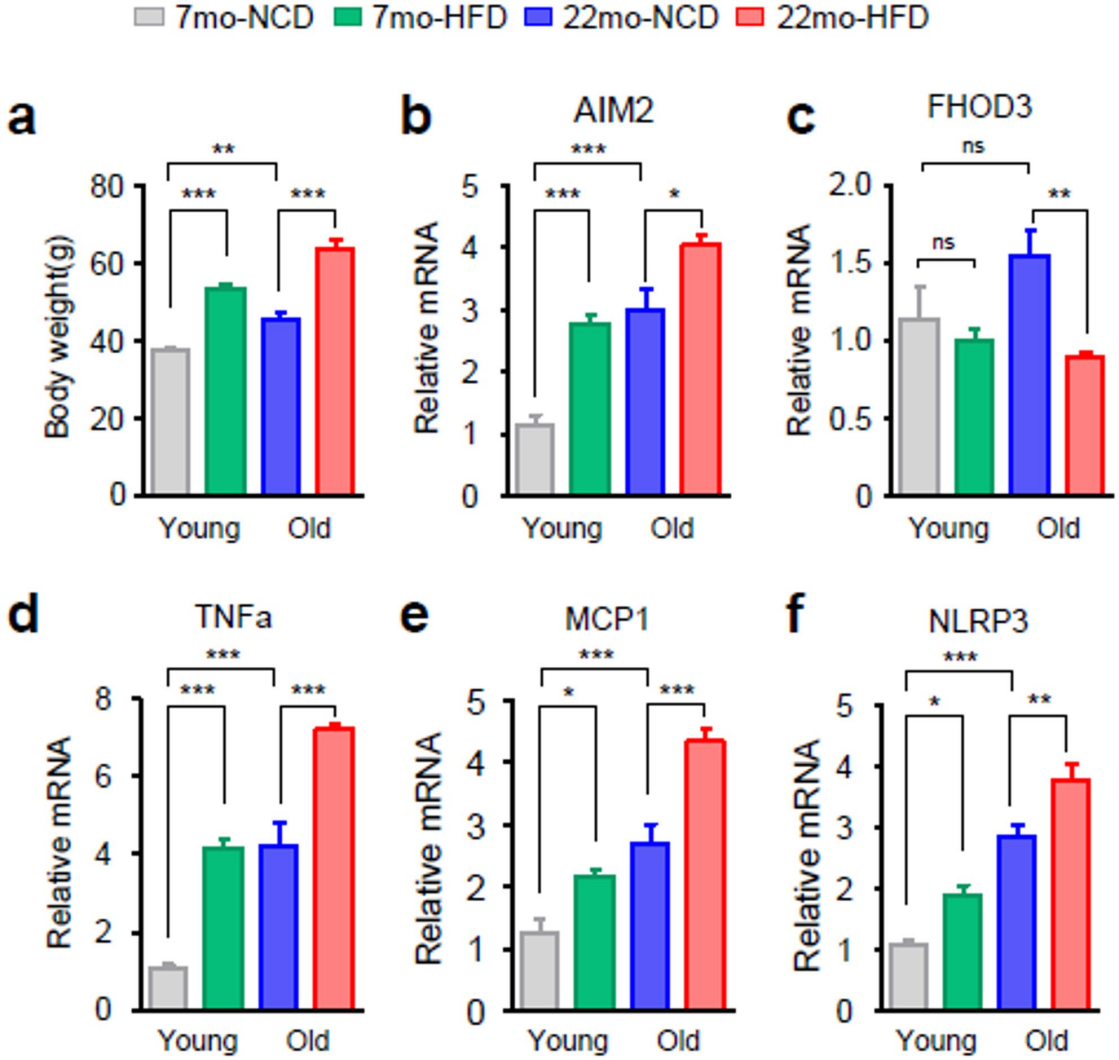
Validation of T2D biomarkers in white adipose tissue of aged mice. (**A**) Body weight of young (7-month-old; 7mo) and old (22-month-old; 22mo) mice fed a normal chow diet (NCD) or a high-fat diet (HFD) (**B**–**F**) Relative mRNA expression of Aim2, Fhod3, Tnfα, Mcp1, and Nlrp3 in white adipose tissue. The data are presented as the mean ± SEM values. *p-value < 0.05; ** p-value < 0.01; *** p-value < 0.001; ns, not significant.

According to data from the GEO datasets, AIM2 showed relatively high expression levels in the adipose tissue of older adults with diabetes, while exhibiting lower FHOD3 expression levels than those of the older healthy group. To experimentally corroborate the differential expression of the age-related biomarker genes in T2D, we conducted qPCR analysis on the white adipose tissues of mice. Based on previous report^22^ that high fat diet intake induces metabolic alterations similar to those observed in humans presenting with metabolic syndrome including elevated fasting insulin levels, insulin resistance, and steatosis, we fed adult (3-month-old) and old (18-month-old) C57BL/6 mice a normal chow diet (NCD) and a high fat diet (HFD) for 4 months. As expected, old mice gained more body weight than young mice not only NCD but also HFD (Fig. 8A). The results showed that the levels of Aim2 were substantially greater in the aged mice compared with the young mice fed NCD, while the expression of Fhod3 was not statistically different between the two groups (Fig. 8B,C). In particular, HFD increased the mRNA expression of the Aim2 in both young and aged mice, and the effects were greater in aged mice. However, only aged mice fed a HFD exhibited decreases in mRNA expression of Fhod3. The levels of tumor necrosis factor-alpha (TNF-α), monocyte chemotactic protein-1 (MCP-1) and NOD-like receptor pyrin containing-3 (NLRP3), as known markers of inflammation, were significantly increased in the aged mice compared with the young mice (Fig. 8D–F). Overall, these results collectively indicate that hub genes may be closely associated with regulation of the age-related T2D. To further validate these results in human subject, we analyzed the transcriptome data obtained from the GSE175495, GSE244118, and GSE 107,894 dataset. As shown in Figure S1, hub genes displayed similar expression pattern. Interestingly, the average expression of AIM2 was found to be more abundant in the SAT of old individuals, indicating its potential role in age-dependent adipose tissue malfunction.

## Discussion

T2D is a growing global health concern due to its increasing prevalence, substantial economic burden, and severe complications. Early identification of high-risk individuals and timely intervention are crucial for preventing or delaying T2D onset^23^. Advances in bioinformatics have provided powerful tools for discovering disease-associated biomarker genes^24^. In this study, we used publicly available GEO datasets, analyzed gene expression profiles from SAT, and identified DEGs between older individuals with T2D and those without T2D. To identify robust T2D-associated biomarkers, we applied LASSO regression, SVM-RFE, and RF models. By integrating the results of these three methods, we identified two key biomarker genes, AIM2 and FHOD3. Multiple validation studies confirmed the robustness of these genes in distinguishing older individuals with T2D from those without T2D.

This study revealed that enrichment analysis of the DEGs revealed significant associations with immune and metabolic processes in older adults with diabetes. These DEGs were enriched in immune regulatory pathways, such as cytokine–cytokine receptor interactions, and metabolic pathways, such as steroid hormone biosynthesis and retinol metabolism. Cytokines and their receptors play crucial roles in coordinating immune and inflammatory responses and are involved in the pathogenesis of T2D^25^. In addition, fat tissue in aged obese individuals is an important source of circulating cytokines and chemokines during systemic inflammation, thereby expanding the inflammatory environment within adipose tissue. Recent studies have suggested that aging-associated insulin resistance is related to immunosenescence and inflammaging. These findings highlight the interplay between immune responses and metabolic dysregulation in elderly T2D patients, reinforcing the role of inflammation and metabolic alterations in disease pathogenesis.

A prevalent feature of most age-related diseases is chronic and low-grade systemic inflammation, which has also been observed in older adults as well as in patients with diabetes. Notably, overweight and obese older adults drastically increase chronic low-grade systemic inflammation, hence they are highly susceptible to developing T2D. Healthy adipose tissue contributes to a healthy metabolic environment. With the onset of aging, however, adipose tissue undergoes significant changes, such as reduced adipocyte size, tissue fibrosis, altered lipolysis, and increased angiogenic capacity^26^, which may be related to the insulin-resistant environment. Previous reports have demonstrated that impaired plasticity of subcutaneous white adipose tissue is already evident in middle-aged mice, which may be related to early metabolic complications^27^.

LASSO, SVM-RFE, and RF analyses revealed that, from a set of 210 DEGs in SAT, AIM2 and FHOD3 were key predictive biomarkers for T2D in elderly individuals. Furthermore, the nomogram model and calibration curves confirmed the strong predictive performance and potential clinical applicability of the model. Although our RF algorithm did not detect Chitinase-3 like-protein-1(CHI3L1) with the top 10 genes, we also identified CHI3L1 as another overlapping gene via LASSO regression and the SVM-RFE algorithm. CHI3L1 plays a central role in inflammation and is associated with many diseases such as obesity, diabetes, and liver fibrosis. This is consistent with a previous study in which elevated CHI3L1 levels were associated with insulin resistance in patients with T2D^28^.

In both the training and test sets, AIM2 was expressed at significantly higher levels in the OT2D group than in the OH group, whereas FHOD3 was expressed at higher levels in the OH group and at lower levels in the OT2D group. This differential expression pattern suggests potential gene regulatory changes associated with T2D in elderly individuals. These findings indicate that a predictive model incorporating the SAT biomarkers AIM2 and FHOD3 could serve as a highly reliable and robust method for the accurate identification of T2D in elderly individuals.

AIM2 (absent in melanoma 2) is a protein that detects double-stranded DNA (dsDNA) within cells and has been reported to be associated with inflammatory responses and metabolic diseases^29^. Recent studies on diabetes and AIM2 have shown that AIM2 expression in monocytes and mitochondrial DNA (mtDNA) levels in the serum are elevated in T2D patients. These findings suggest that AIM2 inflammasome activation in macrophages may contribute to insulin resistance and chronic inflammation^4,30,31^. AIM2 also plays a significant role in T2D complications. In diabetic cardiomyopathy, AIM2 promotes reactive oxygen species (ROS) production and induces cardiomyocyte apoptosis. In diabetic kidney disease (DKD), AIM2 may mediate inflammatory responses and AIM2-deficient mice exhibit reduced kidney damage and inflammation, suggesting a potential shared inflammatory mechanism between T2D and chronic kidney disease (CKD)^32^. Furthermore, research findings have indicated that metformin may regulate the AIM2 pathway and alleviate diabetes-related inflammatory responses^33^. Taken together, these findings suggest that AIM2 is closely linked to the development and complications of T2D and may serve as a potential therapeutic target in the future^34,35^.

FHOD3 (formin homology 2 domain-containing 3) is a formin homology protein located on chromosome 18q12.2 that shares conserved domains with FHOD1. FHOD3 produces three isoforms via alternative splicing and plays a role in actin regulation, contributing to organogenesis, tissue homeostasis, and cancer-cell invasion^36^. FHOD3 is involved primarily in sarcomere organization, myofibrillogenesis, and the regulation of cardiomyocyte contractility. Genetic variants of FHOD3 have been linked to hypertrophic cardiomyopathy (HCM), dilated cardiomyopathy (DCM), and an increased risk of cardiovascular mortality^37,38^. FHOD3 also plays a role in heart development and neural tube closure^39^. Given the close relationship between cardiovascular diseases and T2D, FHOD3 may have implications for T2D-related complications. However, there is currently no direct evidence linking FHOD3 to T2D in older adults, and further research is needed to clarify whether FHOD3 has a role in T2D.

Obesity, similar to aging, is associated with inflammation. Adipose tissue macrophages in obesity are burdened with increased adipocyte death, leading to inflammasome activation and the formation of crown-like structures (CLSs) in adipose tissue; these macrophages aggregate around dead adipocytes^3^. A previous study revealed that FHOD3 was downregulated in fat biopsy samples from individuals with CLS compared with those from individuals without CLS, whereas genes involved in the response to inflammation were upregulated in individuals with CLS^40^. Consistent with previous data, our data revealed a lower abundance of FHOD3 in the SAT of older adults with diabetes. Its expression level was found to be associated with age and metabolic status; therefore, we expected that the decrease in FHOD3 expression in adipose tissue would be associated with the development of T2D in older adults.

In this study, two biomarker genes associated with T2D in elderly individuals were identified via LASSO regression, SVM-RFE, and RF, and a potential basis for risk prediction and therapeutic strategies was provided. However, several limitations should be considered. First, the transcriptomic data utilized in this study were obtained from publicly available GEO datasets. Despite applying batch effect correction, inherent variations between datasets may still have influenced the findings. Therefore, additional validation in independent cohorts is essential to increase the robustness and generalizability of these results. Second, gene expression exhibits tissue specificity, and our analysis was confined to SAT. Consequently, the identified biomarkers may not be applicable to other tissues. Future studies should incorporate multiple adipose depots or other metabolically active tissues to evaluate the broader relevance and biological significance of these biomarkers in T2D pathogenesis. Third, this study was a retrospective analysis based on transcriptomic data, limiting the ability to establish causal relationships between the identified biomarkers and T2D. To strengthen the reliability of our findings, future validation for these biomarkers should be prioritized in a clinical cohort to confirm their clinical applicability. Furthermore, experimental validation, including functional assays and mechanistic studies, is necessary to elucidate the precise roles of these genes disease progression to enhance their translational potential as biomarkers. Finally, the predictive model demonstrated strong performance on the basis of bulk RNA-seq data derived from subcutaneous abdominal adipose tissue of older adults with and without T2D. However, owing to the limited availability of suitable external datasets, independent validation could not be performed in this study. As such, the potential risk of overfitting should be acknowledged when these findings are interpreted. To further evaluate the clinical applicability of the identified biomarkers and clarify their role in T2D progression, future studies should prioritize large-scale, multicenter validation using independent cohorts.

## Conclusion

In the SAT of elderly individuals, *AIM2* and *FHOD3* were identified as key biomarker genes for T2DM through transcriptomic analysis and machine learning approaches, including LASSO regression, SVM-RFE, and RF. A greater prevalence of T2D was observed in individuals with abnormal expression of these genes than in those with normal expression, highlighting the potential role of these genes in SAT aging and diabetes pathophysiology. These findings provide insights into the molecular mechanisms linking SAT dysfunction and T2D in elderly individuals. The relevance of these biomarkers in other tissues, such as blood and muscle, should be explored in future studies to assess the systemic involvement of these genes in metabolic aging. The identification of these genes as biomarkers provides a new direction for precision medicine, enabling early diagnosis and targeted therapeutic strategies for aging-related diabetes.

## Supplementary Information

Below is the link to the electronic supplementary material.

Supplementary Material 1

Supplementary Material 2

## Data Availability

The datasets analyzed in the study are available from the corresponding author upon reasonable request.
