# Supplementary material for: Identifying potential biomarkers for type 2 diabetes in the adipose tissue of older adults via multiple machine learning algorithms
Source: Sci Rep. 2025 Dec 29;15:44904. doi: 10.1038/s41598-025-29141-9 (PMC12748723; doi:10.1038/s41598-025-29141-9)

**Supplementary information**

Figure S1. Expression level of AIM2 and FHOD3 changes with aging and metabolically unhealthy obesity in subcutaneous abdominal adipose tissue based on analyzing (a) GSE175495 dataset and (b) GSE244118 dataset. (c) Analysis of GSE107894 data suggested that the expression levels of AIM2 and FHOD3 in subcutaneous abdominal adipose tissue of the older adults treated with or without metformin or placebo for 6 weeks.


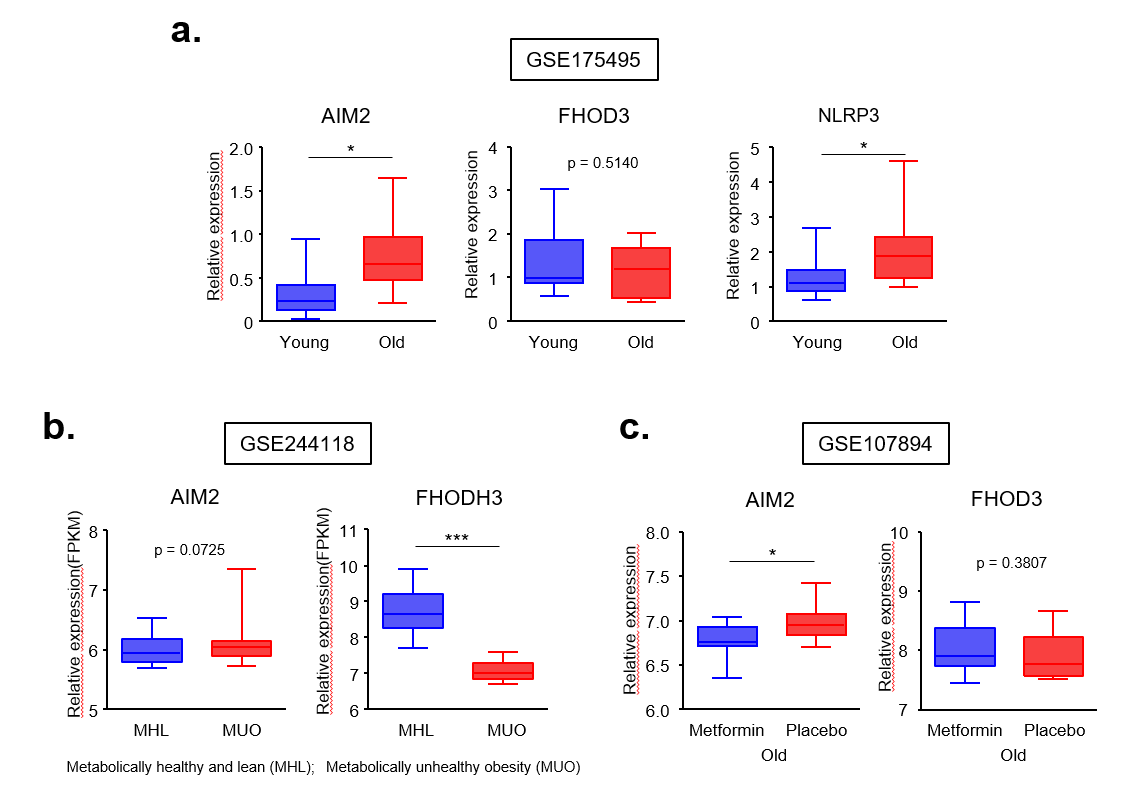

Supplement: Supplementary file 1 — Supplementary Material 1 [file 41598_2025_29141_MOESM1_ESM.docx]
